# Efficacy of *Saccharomyces* yeast postbiotics on cell turnover, immune responses, and oxidative stress in the jejunal mucosa of young pigs

**DOI:** 10.1038/s41598-024-70399-2

**Published:** 2024-08-20

**Authors:** Marcos Elias Duarte, Sung Woo Kim

**Affiliations:** Department of Animal Science, North Carolina State University, Raleigh, NC 27695 USA

**Keywords:** Apoptosis, Cell proliferation, Immunocompetence, Jejunal mucosa, Nursery pigs, *Saccharomyces* yeast postbiotics, Animal physiology, Mucosal immunology

## Abstract

This study aimed to determine the effects of *Saccharomyces* yeast postbiotics on cell turnover, immune responses, and oxidative stress in the jejunal mucosa of pigs. Thirty-two newly weaned pigs at 6.05 ± 0.24 kg were assigned to two dietary treatments based on a randomized complete block design. The treatments were control group receiving a basal diet and a group supplemented with *Saccharomyces* yeast postbiotics (175 g/ton diet) in the basal diet. After 35 d of the study, pigs were euthanized and jejunal mucosa were collected to assess immune status, oxidative stress, barrier markers, cell proliferation, and apoptosis. *Saccharomyces* yeast postbiotics reduced (*P* < 0.05) the fecal score from d 3 to d 7 and tended to increase the gene expression of interferon-γ (IFN-γ) (*P* = 0.071) and mammalian/mechanistic target of rapamycin (mTOR) (*P* = 0.080), decrease the gene expression of B-cell lymphoma 2-associated X protein 1 (BAX1) (*P* < 0.05), tended to decrease the gene expression of serum and glucocorticoid-induced protein kinase 1 (SGK1) (*P* = 0.066), increased (*P* < 0.05) cell proliferation in the crypts, and tended to increase the villus height (*P* = 0.078) and crypt depth (*P* = 0.052) in the jejunum. In conclusion, the supplementation of *Saccharomyces* yeast postbiotics in nursery diets reduced diarrhea within the first week after weaning and provided protection to the villi in the jejunum by enhancing the immune responses of nursery pigs, promoting crypt cell proliferation, and reducing the expression of genes associated with apoptosis without affecting inflammatory and oxidative stress status in the jejunum of the nursery pigs.

## Introduction

Maintaining good health of the small intestine represents a crucial aspect of overall well-being especially in young animals^1^. The jejunum is the major part of the small intestine where food digestion and nutrient absorption mainly occur. Jejunal mucosa integrity is an essential factor for the maintenance of intestinal functions, including digestion, absorption, and protection of health status^2–4^. Under homeostasis, the cell proliferation in the crypt occurs constantly in a balance with the apoptosis to maintain the epithelial integrity in the jejunal mucosa^5,6^. However, a disruption of intestinal homeostasis, caused by different factors, including increased inflammation and oxidative stress, results in excessive apoptosis and reduced expression of tight junction proteins in enterocytes^7,8^. Consequently, the increased apoptosis results in the destruction of villi, negatively impacting the digestive and protective functions of the jejunum^4,9^. Furthermore, stressors, including diarrhea, can be transient and disappear in 5 to 7 days, whereas its impacts on intestinal health have been reported to last for 3 to 4 weeks, even in the absence of clinical symptoms^4,9,10^.

The regenerative capacity of the jejunum is vital to maintain homeostasis of intestinal functions. Immune response, cell proliferation, and apoptosis are regulated by the mammalian target of rapamycin (mTOR) in diverse signaling pathways^11–13^. In the jejunal mucosa, the mTOR can be activated by cytokines and Toll-like receptors (TLR)^14^. Pattern recognition receptors (PRR), including TLR, and nucleotide oligomerization domain (NOD) are proteins that recognizes molecules from microorganisms and are necessary for the interaction with the host^15,16^. The intricate balance among nutrient absorption, immune response, and microbial interactions within the gastrointestinal tract plays a pivotal role in shaping the health of animals^3,15^.

Recent studies have explored the potential of dietary interventions to enhance intestinal health in pigs by reducing inflammation, and oxidative stress, and promoting the integrity of the intestinal epithelium ^9,17–19^. Among these strategies, postbiotics have been used to modulate intestinal microbiota, thereby reducing inflammation and enhancing intestinal morphology^9,19–21^. Yeast-based postbiotics have been widely employed in animal diets to prevent and mitigate the deleterious effects of challenges posed by *Escherichia coli* and mycotoxins^19,20,22,23^. The primary functional compounds in *Saccharomyces* yeast postbiotics are found in the cell wall^24^. Beta-glucans from the yeast cell wall can modulate the intestinal immune response, suppressing TLR4-mediated nuclear factor-kappa B (NF-κB) activation, reducing inflammation, oxidative stress, and damage to the intestinal epithelium^25^. Additionally, a yeast-based postbiotic containing bioactive peptides has been reported to stimulate growth and differentiation through mTOR activation in muscular cells of pigs^26^ and increase crypt depth, an indicator of cell proliferation, in broilers^27^.

Therefore, it was hypothesized that yeast-based postbiotics enriched with bioactive peptides can activate mTOR signaling, enhancing cell turnover and epithelial integrity, improving immune responses, and reducing oxidative stress in the jejunal mucosa of pigs. Thus, the objective of this study was to determine the effects of yeast-based postbiotics on mTOR activation, cell turnover, morphology, immune, and oxidative stress status in the jejunum of pigs.

## Results

### Fecal score

Pigs grew normally during 35 day of the experimental period with initial and final body weights at 6.1 ± 0.2 kg and 21.6 ± 0.7 kg, respectively. Pigs consumed an average of 670 ± 30 g diet gaining 445 ± 16 g body weight per day during the study. When data were analyzed by day, dietary supplementation of *Saccharomyces* yeast postbiotics reduced (*P* < 0.05, close to normal at 3), the fecal score and the incidence of severe diarrhea (11/16 to 6/16) from d 3 to d 7 (Table 1). When data were analyzed by phases, the supplementation of *Saccharomyces* yeast postbiotics tended to reduce (*P* = 0.095, 3.7 to 3.38) the fecal score in phase 1.

**Table 1:** Fecal score of pigs fed diets supplemented with *Saccharomyces* yeast postbiotics.

Item	Treatment^1^			
	Control	Postbiotic	SEM	*P* value
Fecal score (pigs with severe diarrhea/pigs per treatment)^2^				
d 3 to 7	3.89 ± 0.78 (11/16)	3.39 ± 0.53 (6/16)	0.17	0.044
d 8 to 10	3.39 ± 0.59 (2/16)	3.30 ± 0.42 (3/16)	0.22	0.626
d 0 to 10 (Phase 1)	3.70 ± 0.63 (11/16)	3.38 ± 0.39 (6/16)	0.14	0.095
d 11 to 19 (Phase 2)	3.05 ± 0.41 (0/16)	2.99 ± 0.49 (0/16)	0.18	0.700
d 20 to 35 (Phase 3)	3.21 ± 0.40 (0/16)	2.86 ± 0.47 (0/16)	0.18	0.197

^1^Mean ± Standard error (n = 16).

^2^Fecal scores greater than 5 were considered as severe diarrhea.

### Mucosal immune and oxidative stress status

The supplementation of *Saccharomyces* yeast postbiotics in nursery pig diets did not affect the concentration of immune and oxidative stress markers in the jejunal mucosa (Table 2).

**Table 2:** Immune and oxidative stress status of pigs fed diets supplemented with *Saccharomyces* yeast postbiotics.

Item	Treatment^1^			
	Control	Postbiotic	SEM	*P* value
Interleukin 6, pg/mg protein	31.75 ± 3.61	25.87 ± 3.11	7.50	0.165
Interleukin 8, ng/mg protein	0.76 ± 0.08	0.64 ± 0.05	0.08	0.214
Tumor necrosis factor α, pg/mg protein	1.76 ± 0.26	1.96 ± 0.41	0.34	0.682
Malondialdehyde, nmol/mg protein	0.54 ± 0.05	0.57 ± 0.06	0.05	0.740
Protein carbonyl, nmol/mg protein	1.44 ± 0.16	1.51 ± 0.16	0.16	0.771
Immunoglobulin A, µg/mg protein	2.46 ± 0.39	2.01 ± 0.23	0.31	0.316
Immunoglobulin G, µg/mg protein	2.04 ± 0.20	1.94 ± 0.28	0.29	0.770

^1^Mean ± Standard error (n = 16).

### Gene expression of intestinal markers

The dietary treatments did not affect the Ct of the housekeeping gene β-actin (data not shown). The supplementation of *Saccharomyces* yeast postbiotics did not affect the gene expression of markers associated with intestinal barrier functions (Table 3). The supplementation of *Saccharomyces* yeast postbiotics tended to increase the gene expression of interferon-γ (IFN-γ) by 35% from 1.08 to 1.46 (*P* = 0.071), and mTOR by 38% from 1.01 to 1.39 (*P* = 0.080) in the jejunal mucosa of nursery pigs. The supplementation of *Saccharomyces* yeast postbiotics tended to decrease the gene expression of Serum and glucocorticoid-induced protein kinase 1 (SGK1) by 37% from 1.08 to 0.68 (*P* = 0.066) and reduced (*P* < 0.05) the gene expression of B-cell lymphoma 2-associated X protein 1 (BAX1) by 19% from 1.01 to 0.82 in the jejunal mucosa of nursery pigs.

**Table 3:** Relative gene expression of intestinal markers in pigs fed diets supplemented with *Saccharomyces* yeast postbiotics.

Item	Treatment^1^			
	Control	Postbiotic	SEM	*P* value
Claudin-1	1.00 ± 0.18	0.74 ± 0.16	0.19	0.339
Occludin	1.00 ± 0.06	0.98 ± 0.08	0.08	0.839
Zonula Ocludin-1	1.00 ± 0.07	0.94 ± 0.07	0.07	0.593
Mucin 2	1.00 ± 0.10	0.86 ± 0.06	0.09	0.261
Toll-like receptor 2	1.06 ± 0.14	1.39 ± 0.55	0.41	0.537
Toll-like receptor 4	1.18 ± 0.25	0.95 ± 0.13	0.24	0.472
Nucleotide oligomerization domain 1	1.06 ± 0.17	0.72 ± 0.13	0.16	0.174
Nucleotide oligomerization domain 2	1.06 ± 0.13	0.78 ± 0.11	0.12	0.167
Cluster of differentiation 14	1.05 ± 0.12	0.84 ± 0.11	0.15	0.194
Cluster of differentiation 3	1.04 ± 0.11	0.95 ± 0.13	0.12	0.591
Nuclear factor-κB	1.07 ± 0.15	1.05 ± 0.11	0.16	0.931
Interferon-γ	1.08 ± 0.16	1.46 ± 0.15	0.23	0.071
Mammalian/mechanistic target of rapamycin	1.01 ± 0.07	1.39 ± 0.19	0.15	0.080
Notch1	1.01 ± 0.06	1.21 ± 0.14	0.10	0.192
Yes-associated protein 1	1.01 ± 0.05	1.13 ± 0.10	0.08	0.316
Olfactomedin-4	1.05 ± 0.13	0.89 ± 0.15	0.14	0.447
Serum and glucocorticoid-induced protein kinase 1	1.08 ± 0.15	0.68 ± 0.13	0.14	0.066
Myelocytomatosis oncogene	1.03 ± 0.10	0.99 ± 0.13	0.12	0.799
Ornithine decarboxylase	1.01 ± 0.05	1.12 ± 0.13	0.10	0.469
B-cell lymphoma 2-associated X protein 1	1.01 ± 0.05	0.82 ± 0.06	0.05	0.031
Caspase-3	1.03 ± 0.08	0.94 ± 0.05	0.07	0.351

^1^Mean ± Standard error (n = 8).

### Jejunal morphology and crypt cell proliferation

The supplementation of *Saccharomyces* yeast postbiotics tended to increase the villus height by 12% from 438 to 492 µm (*P* = 0.078) and the crypt depth (*P* = 0.052) by 10% from 246 to 270 µm in the jejunum (Table 4). The supplementation of *Saccharomyces* yeast postbiotics increased (*P* < 0.05) the cell proliferation in crypts of the jejunum by 14% from 53.3 to 60.6 units/crypt (as measured by immunohistochemistry identifying the Ki-67^+^ cells counted in a crypt). However, the supplementation of *Saccharomyces* yeast postbiotics in nursery pig diets did not affect the villus width, and the villus height to crypt depth ratio (VH:CD).

**Table 4:** Jejunal morphology of pigs fed diets supplemented with yeast-based postbiotic.

Item	Treatment^1^			
	Control	Postbiotic	SEM	*P* value
Villus height, µm	438 ± 20	492 ± 22	22	0.078
Villus width, µm	104 ± 2	100 ± 2	3	0.310
Crypt depth, µm	246 ± 7	270 ± 10	10	0.052
VH:CD^2^	1.76 ± 0.11	1.81 ± 0.12	0.11	0.779
Ki-67^+3^, unit	53.3 ± 2.9	60.6 ± 1.8	3.01	0.038

^1^Mean ± Standard error (n = 16).

^2^Villus height to crypt depth ratio.

^3^Number of positive crypt cell proliferation in jejunum.

## Discussion

A healthy intestinal mucosa plays a fundamental role in efficient nutrient absorption and prevents increased intestinal permeability^2^. In addition, the of intestinal mucosa is the first line of defense against pathogens invasion and its integrity is an essential factor for the maintenance of intestinal functions, including immune responses^8,28,29^. Along the gastrointestinal tract, the jejunum can be considered a key portion of analyzing the interaction among dietary compounds, microbiota, and the immune system^15^.

In this this study, the fecal score and the incidence of severe diarrhea were recorded to evaluate the overall health status of the pigs during the experimental period. Fecal score is a parameter largely used to evaluate the digestive health in animal models^9,30–33^. In pig production, weaning is a stressful event that makes pigs more susceptible to diarrhea incidence, mainly due to the disruption of the intestinal microbiota^34^. Studies have reported that the effects of pathogenic infection, including enterotoxigenic *Escherichia coli*, on intestinal health parameters can last for up to 21 days after the inoculation, even in the absence of clinical symptoms which ceases around 4 to 11 days^4,9,10^. The change from milk to solid and a less digestible plant-based diet is the major factor altering intestinal microbiota, increasing inflammation, and oxidative damage in the intestinal mucosa^33,35^. When the yeast postbiotic was supplemented in nursery diets, it reduced the fecal score to normal and reduced the incidence of severe diarrhea (pigs with fecal score greater than 5) during the first 7 days post-weaning (Table 1). The reduced fecal score observed in this study might be attributed to increased IFN-γ and mTOR expressions in the postbiotic group. The improved immune responses, indicated by the increased expression of IFN-γ and mTOR, may have led to improved defense mechanisms against pathogenic bacteria, reducing inflammation in jejunal mucosa, although the gene expression of PRR, including TLR2, TLR4, NOD1, NOD2, CD14, and CD3, did not differ between treatments (Table 3).

During inflammation in response to a pathogen, the host produces nitric oxide (NO) as an antimicrobial^36^. The NO is rapidly oxidized to form nitrate that in conjunction with other reactive oxygen species (ROS) causes oxidative stress^37,38^. Under homeostasis, a complex of antioxidant enzymes, including superoxide dismutase (SOD), catalase (CAT), and glutathione peroxidase (GPx), eliminates the ROS reducing the oxidative damages^38^. Excessive ROS generation disrupts the cellular redox balance, leading to the accumulation of oxidative damage products, including protein carbonyls and malondialdehyde (MDA), highly stable markers of oxidative stress^39,40^. Although in this study, the oxidative stress markers were not affected by the treatments, the carbonylation significantly alters protein structure and function, triggering apoptosis pathways^41,42^.

In the current study, the use of yeast postbiotics in a nursery diet reduced the gene expression of BAX1 in jejunal mucosa. BAX1 is a protein that triggers apoptosis by binding and blocking the apoptosis inhibitor BCL2^43,44^. Under stress, BAX1 undergoes a conformational shift, driving its relocation to the mitochondrial membrane. This disrupts the membrane, causing the release of cytochrome c, a key player in initiating programmed cell death that activates caspase-3 (CASP3)^43,45^. Interestingly, the expression of CASP3 was not affected by the treatments in this study, possibly because CASP3 is expressed in its inactive form and is activated when it receives a cell death stimulus^46^.

The reduced expression of BAX1 reported in the current study in pigs fed diets supplemented with yeast postbiotic can be associated with the increased expression of mTOR observed in the same group. mTOR impairs the degradation of myeloid cell leukemia 1 (MCL-1), a protein that prevents apoptosis^47^. However, it is important to note that mTOR can both inhibit and induce apoptosis. mTOR can also induce p53 to promote BAX translocation to the mitochondrial membrane stimulating apoptosis^48^.

The reduced expression of BAX1 and the increased expression of mTOR and IFN-γ seem to have conferred enhanced protection of villi in the jejunum of pigs fed yeast postbiotic. The villus height showed a trend to increase in pigs fed yeast postbiotic. The increased villus height can be a reflection of the increased number of ki67^+^ cells in jejunal crypt. Ki67 is a marker of cell proliferation associated with activation of mTOR in cancer cells^49,50^. Previous studies using the same product have shown that the yeast-based postbiotic containing bioactive peptides can stimulate growth and differentiation through mTOR activation in muscular cells of pigs^26^ and increase crypt depth, an indicator of cell proliferation, in broilers^27^. These results indicate that the yeast postbiotic influences intestinal health by protecting villi by reducing diarrhea and cell apoptosis, increasing cell proliferation and immune responses of the jejunal mucosa.

In conclusion, the supplementation of *Saccharomyces* yeast postbiotics, at 175 g/ton of diet for nursery pigs reduced the diarrhea incidence within the first week after weaning and provided protection to the villi in the jejunum by enhancing the immune responses of nursery pigs, promoting crypt cell proliferation, and reducing the expression of genes associated with apoptosis without affecting inflammatory and oxidative stress status in the jejunum and the growth of nursery pigs.

## Methods

The experimental protocol was approved (Approval number 22-438) by the Institutional Animal Care and Use Committee of North Carolina State University. All methods were performed in accordance with the relevant guidelines and regulations. The experiment is reported in accordance with ARRIVE guidelines (https://arriveguidelines.org).

### Animals, experimental design, and diets

The experiment was conducted at the Swine Educational Unit at North Carolina State University (Raleigh, NC). Thirty-two newly weaned pigs at 21 d of age (16 barrows and 16 gilts) with initial BW of 6.05 ± 0.24 kg were purchased from a commercial farm (NG Purvis Farms, Robbins, NC, USA) and allotted into two dietary treatments based on a randomized complete block design with initial BW and sex as blocks. The dietary treatments were a control (basal diet) and Postbiotic (basal diet with *Saccharomyces* yeast postbiotics containing bioactive peptides at 175 g/ton diet). *Saccharomyces* yeast postbiotics (celluTEIN) was obtained from Puretein Bioscience LLC (Minneapolis, MN, USA) and are available as a commercial item. The amount of supplementation was previously evaluated by Kim and Duarte^49^.

The Postbiotic was supplemented replacing corn in the basal diet, formulated meeting the nutrient requirements of NRC (2012), as are shown in Table 5. Pigs were housed individually in a pen and had ad libitum access to diets and water. The experimental period was 35 d, which was divided into 3 dietary phases: phase 1 (d 1 to d 10), phase 2 (d 10 to d 19), and phase 3 (d 19 to d 35) to provide nutrients meeting the requirement. Pigs and the diet disappearance were individually weighed by the end of each phase to monitor diet intake and growth of pigs. Fecal score was recorded bi-day from d 3 of experiment. Fecal scores were evaluated daily by the same person considering the following scale: very hard and dry stool (score 1), firm stool (score 2), normal stool (score 3), loose stool (score 4), and watery stool with no shape (score 5) as described by Cheng et al.^50^.

**Table 5:** Composition of basal diets (as-fed basis).

Item	Phase 1	Phase 2	Phase 3
Ingredient, %			
Corn, yellow	41.96	49.52	60.57
Whey permeate	19.00	13.00	6.00
Soybean meal	18.50	23.50	28.50
Poultry meal	9.00	5.00	0.00
Fish meal	5.00	3.00	0.00
Hydrolyzed soybean meal	3.00	1.50	0.00
Poultry fat	1.10	1.70	1.42
L-lysine HCl	0.58	0.47	0.46
L-methionine	0.27	0.19	0.16
L-threonine	0.20	0.14	0.14
L-tryptophan	0.03	0.01	0.00
L-valine	0.02	0.01	0.03
Dicalcium phosphate	0.00	0.38	0.95
Limestone	0.44	0.68	0.87
Vitamin premix^1^	0.03	0.03	0.03
Mineral premix^2^	0.15	0.15	0.15
Salt	0.22	0.22	0.22
Calculated composition:			
Dry matter, %	90.8	90.3	89.5
ME, kcal/kg	3,401	3,400	3,351
CP, %	24.4	22.3	19.5
SID^3^ Lys, %	1.50	1.35	1.23
SID Met + Cys, %	0.82	0.74	0.68
SID Trp, %	0.25	0.22	0.20
SID Thr, %	0.88	0.79	0.73
SID Val, %	0.95	0.87	0.78
Ca, %	0.85	0.80	0.70
STTD^4^ P, %	0.45	0.40	0.33
Total P, %	0.70	0.64	0.58

^1^The vitamin premix provided the following per kilogram of complete diet: 6,613.8 IU of vitamin A as vitamin A acetate, 992.0 IU of vitamin D3, 19.8 IU of vitamin E, 2.64 mg of vitamin K as menadione sodium bisulfate, 0.03 mg of vitamin B12, 4.63 mg of riboflavin, 18.52 mg of D-pantothenic acid as calcium pantothenate, 24.96 mg of niacin, and 0.07 mg of biotin.

^2^The trace mineral premix provided the following per kilogram of complete diet: 4.0 mg of Mn as manganous oxide, 165 mg of Fe as ferrous sulfate, 165 mg of Zn as zinc sulfate, 16.5 mg of Cu as copper sulfate, 0.30 mg of I as ethylenediamine di-hydroiodide, and 0.30 mg of Se as sodium selenite.

^3^SID, standardized ileal digestible.

^4^STTD P, standardized total tract digestible phosphorus.

### Sample collection

After 35 d of the study, pigs were euthanized by exsanguination after a penetrating captive bolt to head. Mucosal samples (from 15 cm, in 3 aliquots) from mid-jejunum (3 m after the duodeno-jejunal junction) were scraped, placed in Eppendorf tubes (2 mL) and later stored at −80 ℃ (after snap-freezing in liquid nitrogen, immediately after collection) for immune and oxidative stress measurements. Jejunal tissues (1 piece, 3 cm) from the mid-jejunum was collected to measure the expression of gene associated with intestinal barrier markers: Claudins, Occludens, Zonula Occludens-1 (ZO-1), and Mucin 2 (MUC2); as indicators of PRR: TLR2, TLR4, NOD1, NOD2, CD3, and CD14; immune response: NF-κB and IFN-γ; apoptosis and cell proliferation and differentiation: CASP3, BAX1; mTOR, Notch1, YAP1, olfactomedin-4, SGK1, MYC, and ornithine decarboxylase. Jejunal tissue was fixed in 10% formalin for further immunohistochemistry and histological analysis.

### Oxidative stress and immune status

The concentrations of total protein (Pierce BCA Protein Assay Kit #23227, Thermo Fisher Scientific Inc., Rockford, IL, USA), interleukin 6 (Porcine IL-6 DuoSet ELISA, #DY686, R&D System Inc. Minneapolis, MN, USA), IL-8 (Porcine IL-8/CXCL8 DuoSet ELISA, #DY535, R&D System Inc.), tumor necrosis factor-alpha (Porcine TNF-alpha DuoSet ELISA, #DY690B, R&D System Inc.), protein carbonyl (OxiSelect Protein Carbonyl ELISA Kit, #STA-310, Cell Biolabs, Inc. San Diego, CA, USA), MDA (OxiSelect TBARS Assay Kit, #STA-330, Cell Biolabs, Inc.), immunoglobulin G (Pig IgG ELISA Kit, #E101-104, Bethyl Laboratories, Inc., Montgomery, TX), and IgA (Pig IgA ELISA Kit, #E101-102, Bethyl Laboratories, Inc.) were measured by the colorimetric method using commercially available kits according to instructions of the manufacturers. The absorbance was read using a plate reader (Synergy HT, Biotech Instruments, Winooski, VT) and the software (Gen5 Data Analysis Software, BioTek Instruments). The concentration was calculated based on the standard curve created from concentration and absorbance of the respective standards. All the values were within the respective assay range. The analyte concentrations were normalized using the concentration of total protein within each sample and the data were expressed as unit/mg of protein^51^.

### Gene expression of intestinal markers

The RNA extraction from mid-jejunal tissue involved the use of Trizol reagent (Thermo Fisher Scientific Inc.). Subsequently, 1 µg of total RNA underwent complementary DNA synthesis using oligo dT and M-MLV Reverse Transcriptase (Thermo Fisher Scientific Inc.), following manufacturers’ instructions. Briefly, quantitative real-time polymerase chain reaction (PCR) was employed to measure relative messenger ribonucleic acid (mRNA) levels, utilizing Applied Biosystems SYBR Green PCR Master Mix (Thermo Fisher Scientific Inc.) and a QS5 Real-Time PCR System. Results were expressed relative to the housekeeping gene β-actin. Importantly, the Ct of the housekeeping gene β-actin remained unaffected by dietary treatment. Normalization of gene expression to β-actin occurred using the delta–delta–Ct method, as previously described^52^, and results were expressed relative to β-actin levels. All primers (Table 6) were rigorously validated for melting curve, efficiency (100% ± 10%), and linearity (r2 ≥ 0.99) of amplification.

**Table 6:** Sequence of primers for immune responses and barrier function in jejunum.

Gene	Prime	Sequence	Accession number	Size
Claudin 1	Forward	AAACCGTGTGGGAACAACCA	NM_001244539.1	196
	Reverse	CACATGAAAATGGCTTCCCTC		
Occludin	Forward	CAGGCTGCGGTGAGAAGATT	XP_005672579.1	169
	Reverse	TATGTCGTTGCTGGGTGCAT		
Zonula Ocludin-1	Forward	CAGAGACCAAGAGCCGTCC	XM_003480423.4	105
	Reverse	TGCTTCAAGACATGGTTGGC		
Mucin 2	Forward	CAACGGCCTCTCCTTCTCTGT	XM_021082584.1	70
	Reverse	GCCACACTGGCCCTTTGT		
Toll-like receptor 2	Forward	GGGCTGCGTTCATTCATCAG	XM_005653576.3	132
	Reverse	CTGCAGAGGATGGATGGCAA		
Toll-like receptor 4	Forward	CGTGCAGGTGGTTCCTAACA	NM_001113039.2	326
	Reverse	GGTTTGTCTCAACGGCAACC		
NOD^1^1	Forward	AACACCGATCCAGTGAGCAG	NM_001114277.1	230
	Reverse	AAATGGTCTCGCCCTCCTTG		
NOD2	Forward	GTGCCTCCCCTCTAGACTCA	NM_001105295.1	191
	Reverse	ACGAACCAGGAAGCCAAGAG		
Cluster of differentiation 14	Forward	CCCTGCCAAATAGACGACGA	NM_001097445.2	299
	Reverse	TCGAGCGTCAGTTCCTTGAG		
Cluster of differentiation 3	Forward	GTGGATCTGATGGCAGTGGT	NM_214227.1	205
	Reverse	TCCGGATGGGCTCATAGTCT		
Nuclear factor-κB	Forward	GCTGGAATGAAGCACGGAAC	NM_001048232.1	236
	Reverse	GCAAGTTGCATGGCCTTCTC		
Interferon-γ	Forward	GGCCATTCAAAGGAGCATGG	HQ026021.1	119
	Reverse	AAGCTCATCTCACCGGAATTT		
mTOR^2^	Forward	TCTCTATCAAGTTGCTGGCCG	XM_003127584.6	135
	Reverse	CTAGCGCTGCCTTTCGAGAT		
Notch1	Forward	GTGCTTTGGTGTGCTCTTCG	XM_021081037.1	102
	Reverse	CGACGTAACACTGAACCCCA		
Yes-associated protein 1	Forward	CCGACAGGCCAGTACTGATG	XM_021062706.1	131
	Reverse	AGACTACTCCAGTGGGGGTC		
Olfactomedin-4	Forward	CGTTCCAGGTGCCAGTAAGA	XM_003482903.4	94
	Reverse	CCCTGAAAGAAACCTGGGCT		
SGK^3^1	Forward	GCCTCCAGAAACGCATCACC	XM_021078492.1	187
	Reverse	TCCATCATTGCTCCGACACG		
Myelocytomatosis oncogene	Forward	TCCGGAGTGAAAGAGGGTCT	NM_001005154.1	113
	Reverse	AGTGGGCAAAGTTTCGTGGA		
Ornithine decarboxylase	Forward	TGAGTAGGCTGGCACAGATG	XM_021085889.1	128
	Reverse	AGTAGCATGGTTGCAAGGTGA		
BAX^4^1	Forward	GGATAACGGAGGCTGGGATG	XM_021099593.1	147
	Reverse	TTATGGCCCAGATAGGCACC		
Caspase-3	Forward	GGATTGAGACGGACAGTGGG	GCF_000003025.6	124
	Reverse	CCGTCCTTTGAATTTCGCCA		
β-actin	Forward	CAAATGCTTCTAGGCGGACTGT	XM_003124280.5	75
	Reverse	CAAATGCTTCTAGGCGGACTGT		

**Figure 1:**
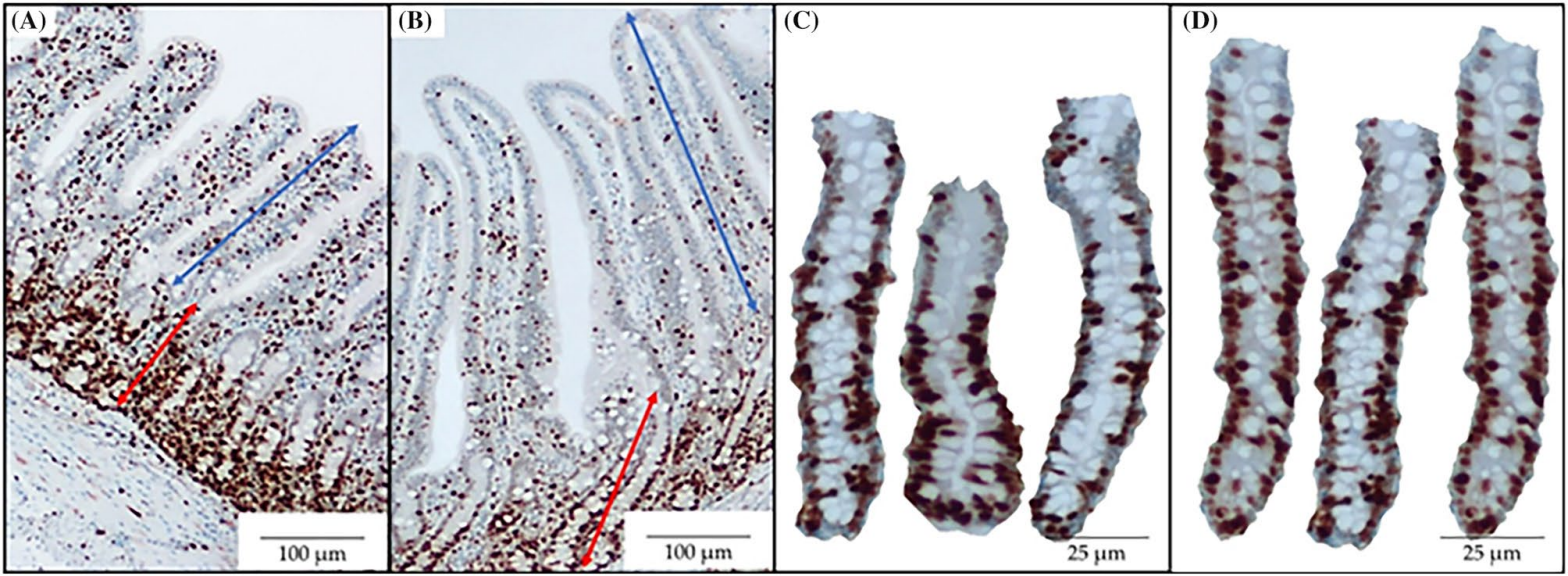
Representative images of immunohistochemistry (Ki67) staining for jejunal morphology and crypt cell proliferation were obtained. Ten images at 40 × magnification of well-oriented villi and their associated crypts ((**A**): Control; (**B**): Postbiotic) were acquired for measuring villus height (from the top to the base of the villus, as indicated with a double arrow line in blue) and crypt depth (from the base of the villus to the bottom of the crypt, as indicated with a double arrow line in red). Ten images at 100 × magnification of the crypts ((**C**): control; (**D**): Postbiotic) were captured for counting the Ki67 + staining cells as an indicator of crypt cell proliferation.

Crypt depth ratio (Fig. 1A–D), as previously described by Deng et al.^53^.

^1^Nucleotide oligomerization domain.

^2^Mammalian/mechanistic target of rapamycin.

^3^Serum and glucocorticoid-induced protein kinase.

^4^B-cell lymphoma 2-associated X protein.

### Jejunal morphology and crypt cell proliferation

Two sections of fixed mid-jejunum tissues were sent to the Lineberger Comprehensive Cancer Center, School of Medicine at the University of North Carolina (Chapel Hill, NC) to be processed and stained with the ki-67 immunohistochemistry assay following their internal protocol. The slides were used to measure the proportion of proliferating cells in the crypt and jejunal villus height, crypt depth, and villus height to crypt depth ratio (Fig. 1A–D), as previously described by Deng et al.^53^.

### Statistical analysis

The experimental unit was pig individually housed and fed. The number of experimental units was pre-determined based on power test^54^. Data were analyzed based on a randomized block design using the Proc Mixed of SAS 9.4 software (SAS Inc., Cary, NC, USA). Dietary treatments were defined as fixed effects and the blocks (sex and initial BW) were the random effects. The data related to diarrhea incidence were analyzed using the Proc Freq of SAS 9.4. The means were calculated using the lsmeans statement in SAS 9.4 and reported with the standard error of the mean (SEM) within each treatment and the SEM related to all values. Statistical differences were considered significant with *P* < 0.05 and tendency with 0.05 ≤ *P* < 0.10.”

## Data Availability

The datasets used and/or analysed during the current study available from the corresponding author on reasonable request.
